# Breastfeeding at discharge or transfer from the maternity hospital: results from the German Perinatal Statistics 2021

**DOI:** 10.1186/s13006-026-00862-5

**Published:** 2026-06-23

**Authors:** Anna-Kristin Brettschneider, Michael Abou-Dakn, Alfred Längler, Andrea Schlune, Regina Ensenauer

**Affiliations:** 1 Department of Nutritional Behaviour, Max Rubner-Institut, Federal Research Institute of Nutrition and Food, Haid-und-Neu-Straße 9, 76131 Karlsruhe, Germany; 2 Clinic for Gynecology and Obstetrics, St. Joseph-Krankenhaus Berlin-Tempelhof, Berlin, Germany; 3 Department of Pediatrics, Gemeinschaftskrankenhaus Herdecke, Herdecke, Germany; 4 Faculty of Health, Professorship for Integrative Pediatrics, University of Witten/Herdecke, Witten, Germany; 5 Executive Body National Breastfeeding Committee, Max Rubner-Institut, Federal Research Institute of Nutrition and Food, Karlsruhe, Germany; 6 The National Breastfeeding Committee at the Max Rubner-Institut, Karlsruhe, Germany

**Keywords:** Neonatal nutrition, Breastfeeding, Breastfeeding at discharge or transfer from maternity hospital, Risk factors, Germany

## Abstract

**Background:**

Given the low proportion of exclusively breastfed children in Germany, breastfeeding promotion is necessary. Beginning in 2021, the nationwide mandatory quality assurance procedure for perinatal medicine in hospitals has routinely collected information on the newborn’s nutrition at hospital discharge or transfer. Aims were to analyze the proportions of newborns either exclusively or partially fed with human milk, or exclusively formula-fed at hospital discharge or transfer and to identify pre- and perinatal factors potentially relevant to the newborn’s nutrition.

**Methods:**

In 2021, nutrition data at hospital discharge or transfer were available from 656,907 newborns. Prevalence rates (%) and 95% confidence intervals were calculated.

**Results:**

At hospital discharge or transfer, 75.4% of newborns were exclusively fed with human milk, 18% partially, and 6.6% were exclusively formula-fed. Newborns exposed to pre- and perinatal risk factors including maternal overweight/obesity, multiple pregnancy, or cesarean section had lower rates of being exclusively fed with human milk. Proportions of newborns exclusively fed with human milk showed regional variations, with the highest rate observed in the federal state of Berlin.

**Conclusion:**

Data from the German Perinatal Statistics suggest that, while more efforts are needed to successfully increase the rates of exclusive breastfeeding in hospitals, certain groups of women could particularly benefit from targeted measures. The data also provide a valuable foundation for breastfeeding monitoring, health reporting, as well as future research examining associations between specific variables and newborn feeding outcomes and for the development of evidence-based breastfeeding support policies.

**Supplementary Information:**

The online version contains supplementary material available at 10.1186/s13006-026-00862-5.

## Introduction

Breastfeeding is the infant’s natural nutrition and provides beneficial short- and long-term health effects for both infant [1–6] and mother [7, 8]. The World Health Organization (WHO) recommends exclusive breastfeeding for the first six months of life, followed by partial breastfeeding with appropriate complementary foods up to the age of two years or beyond [9–11]. In Germany up to 2026, expert-based advice has comprised exclusive breastfeeding until four to six months of age and subsequent continued breastfeeding after introduction of complementary foods [12].

Nevertheless, the initial rate of exclusively breastfed children in Germany was only 68% according to data from the German Health Interview and Examination Survey for Children and Adolescents Wave 2 (KiGGS Wave 2; 2014-17) [13]. Within the first six months of life, the proportion of exclusively breastfed children decreased rapidly: by the second month, it was just over half (57%), by the fourth month, it was 40% and by the sixth month 13%. Meanwhile, the need for breastfeeding promotion has been recognized, and the international research project “Becoming Breastfeeding Friendly” (BBF) [14, 15] was conducted by the Healthy Start – Young Family Network at the Federal Center of Nutrition (BZfE) and the German National Breastfeeding Committee in cooperation with the Yale School of Public Health [16]. As a key recommendation, the need for a German National Strategy for the Promotion of Breastfeeding was stated, and the Max Rubner-Institut (MRI) was commissioned to coordinate the development and implementation of the strategy [17, 18]. Continuous regular and systematic collection of population-wide breastfeeding data does not yet exist in Germany at the federal level, but is essential to quantify the success of measures for breastfeeding promotion.

Therefore, the implementation of a comprehensive system to gather breastfeeding data in Germany is needed, pursuing several approaches, each targeting different stages of breastfeeding as already suggested in 2009 by the National Breastfeeding Committee in Germany [17–19]. One data source is the nationwide mandatory quality assurance procedure for perinatal medicine in hospitals, which also addresses feeding practices at the newborn´s discharge or transfer from the maternity hospital (Perinatal Statistics). Information on the newborn’s nutrition at that time considering exclusive or partial feeding with human milk as well as exclusive feeding with formula were collected for the first time in 2021 [20]. Since the vast majority of deliveries in Germany occur in hospitals (2021: 96.5% of *n* = 795,492 livebirths [20, 21]), these data are an important component of breastfeeding monitoring in Germany.

The aim was to analyze a large set of data from the German Perinatal Statistics 2021 for the proportion of children exclusively or partially fed with human milk, or exclusively fed with formula at hospital discharge or transfer as well as to identify pre- and perinatal factors that might influence the newborn’s nutrition in the first days of life.

## Methods

### Data source and study population

Data from quality assurance procedures pursuant to section 136 of the German Social Code, Book Five (Sozialgesetzbuch, SGB V) of the Federal Joint Committee (Gemeinsamer Bundesausschuss, G-BA) were used for this study [22]. On behalf of G-BA, the Institute for Quality Assurance and Transparency in Healthcare (IQTIG) carries out quality assurance procedures to measure and improve the quality of medical care in Germany [23], and data are made available for secondary scientific purposes [22]. Various aspects of the process and result quality related to adequate perinatal care are recorded by hospital staff on a documentation form using quality indicators and key figures [24]. These data, the so-called German Perinatal Statistics, contain information on the mother (e.g., body mass index (BMI) at first antenatal visit), delivery (e.g., mode of delivery), and the newborn [20]. A panel of experts at federal level in perinatal medicine has been working on the operationalization of (a) quality indicator(s) for breastfeeding, and a corresponding data field has been included in 2021 for the first time [25].

### Outcome variables on nutrition of the newborn at hospital discharge/transfer

Data on the newborn’s nutrition at the time of hospital discharge or transfer were collected using the following categorical outcome variables: (1) “exclusively fed with human milk”, (2) “partially fed with human milk”, and (3) “exclusively fed with formula”. For this analysis, “exclusively fed with human milk” was considered equivalent to “exclusive breastfeeding” as defined by the WHO, meaning the infant received only breast milk (including expressed or pumped milk) and no other foods or liquids, except for medicines or vitamins [26]. “Partially fed with human milk” was considered equivalent to partial breastfeeding and “exclusively fed with formula” to non-breastfeeding.

### Covariates

Prevalence rates for the three outcome variables on the newborn’s nutrition at hospital discharge or transfer were stratified according to the following covariates: maternal BMI at first antenatal visit, maternal age, parity, multiple pregnancy, maturity status, delivery mode, federal state of the maternity hospital, newborn’s sex, length of hospital stay, and transfer to children’s hospital.

### Definitions and categorizations of covariates

*Maternal BMI* (kg/m^2^) at first antenatal visit was calculated based on her self-reported weight before conception (as documented in the maternity log) divided by the square of body height. BMI groups were defined according to the WHO categories: underweight (BMI < 18,5 kg/m^2^), normal weight (BMI 18,5 - < 25 kg/m^2^), overweight (BMI 25 - < 30 kg/m^2^), or obesity (BMI ≥ 30 kg/m^2^) [27]. *Maternal age* at the time of admission to hospital was used and categorized into the following age groups: < 20 years, 20–24 years, 25–29 years, 30–34 years, 35–39 years, or ≥ 40 years. *Parity* was classified based on the number of deliveries. Primiparity was defined as the mother’s first delivery, whereas multiparity referred to the mother’s second or subsequent delivery, in accordance with the German Perinatal Statistics. As the data are collected exclusively in maternity hospitals, nulliparous women are not included in the dataset. *Multiple pregnancies* were categorized as yes or no (singleton birth). *Maturity status* was defined according to gestational age as term (≥ 37 + 0 weeks of gestation) or preterm (< 37 + 0 weeks of gestation). *Delivery mode* was categorized as cesarean section or no cesarean section. *Length of hospital stay* refers to the newborn and was classified according the categories: 1 day or shorter, 2–3 days, 4–6 days, or > 6 days. *Transfer to children’s hospital* was recorded as yes or no. *Newborn’s sex* was classified according to the categories: male, female, or unknown/diverse.

### Statistical analyses

Descriptive analyses were conducted, and mean prevalence rates (%) with 95% confidence intervals are shown. The statistical software package R Version 4.2.2 was used.

## Results

**Fig. 1:**
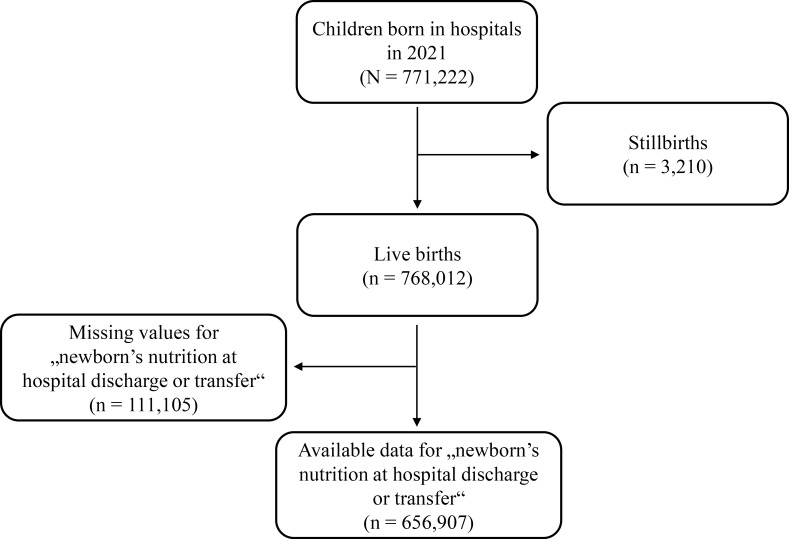
Participant flow chart

For 2021, data from a total of 771,222 mother-child pairs from 655 hospitals were available from the German Perinatal Statistics (Fig. 1). After excluding stillbirths (*n* = 3,210) and cases with missing values for the outcome variables on nutrition (*n* = 111,105), a total of 656,907 newborns, corresponding to 85.2% of all births in hospitals in Germany in 2021, were included in the analysis.

Characteristics of the study population are presented in Table 1. Most mothers had normal weight and were 30–34 years and multiparous. About two-thirds of newborns had been delivered vaginally, and a similar proportion had a hospital stay of 2–3 days. Among all newborns, 75.4% (*n* = 495,002) were exclusively fed with human milk, 18.0% (*n* = 118,238) were partially fed with human milk, and 6.6% (*n* = 43,667) were exclusively fed with formula at hospital discharge or transfer.

**Table 1:** Basic characteristics of the study population (*n* = 656,907)

	*n*	%
**Maternal BMI at first antenatal visit**		
Underweight	20,350	3.1
Normal weight	329,959	50.2
Overweight	152,710	23.2
Obesity	110,196	16.8
Missing	43,692	6.7
**Maternal age at hospital admission**		
< 20	9,480	1.4
20–24	57,090	8.7
25–29	161,666	24.6
30–34	256,043	39.0
35–39	141,178	21.5
≥ 40	31,450	4.8
**Parity**		
Multiparity	416,052	63.3
Primiparity	240,855	36.7
**Multiple pregnancy**		
Yes	19,724	3.0
No	637,183	97.0
**Maturity status**		
Term	615,792	93.7
Preterm	41,115	6.3
**Delivery mode**		
Cesarean section	209,308	31.9
No cesarean section	447,599	68.1
**Federal state of the hospital**		
Baden-Württemberg	101,679	15.5
Bavaria	101,628	15.5
Berlin	37,523	5.7
Brandenburg	11,580	1.8
Bremen	7,584	1.2
Hamburg	23,768	3.6
Hesse	49,582	7.5
Lower Saxony	56,603	8.6
Mecklenburg-Western Pomerania	10,217	1.6
North Rhine-Westphalia	149 067	22.7
Rhineland-Palatinate	29,377	4.5
Saarland	8,984	1.4
Saxony	25,898	3.9
Saxony-Anhalt	13,497	2.1
Schleswig-Holstein	17,226	2.6
Thuringia	12,694	1.9
**Sex (child)**		
Male	336,249	51.2
Female	320,510	48.8
Unknown/diverse	148	0.02
**Length of hospital stay**		
≤ 1 day	121,153	18.4
2–3 days	438,479	66.7
4–6 days	90,327	13.8
> 6 days	6,948	1.1
**Transfer to children’s hospital**		
Yes	60,422	9.2
No	596,485	90.8

BMI, Body mass index

Prevalence rates for the newborn’s nutrition at hospital discharge or transfer differed according to pre- and perinatal characteristics (Table 2). Mothers with overweight or obesity less often exclusively fed their child with human milk and more often exclusively fed their child with formula than mothers with normal weight. Rates also differed according to maternal age at hospital admission: mothers in the age groups < 20 years and 20–24 years less often exclusively fed their child with human milk and more often exclusively fed their child with formula than mothers of older age. The prevalence rate of exclusively feeding formula was higher among women with more than one child, whereas the rate of feeding partially human milk was higher among primiparous women. Women with multiple pregnancies less often exclusively but more often partially fed their child with human milk or exclusively fed with formula compared to women with a singleton birth.

**Table 2:** Prevalence rates for the newborn’s nutrition at hospital discharge or transfer according to pre- and perinatal characteristics

	Exclusively fed with human milk			Partially fed with human milk			Exclusively fed with formula		
	*n*	%	95% CI	*n*	%	95% CI	*n*	%	95% CI
**Maternal BMI at first antenatal visit**									
Underweight	15,550	76.4	(75.8–77.0)	3,437	16.9	(16.4–17.4)	1,363	6.7	(6.4-7.0)
Normal weight	257,265	78.0	(77.8–78.1)	55,617	16.9	(16.7–17.0)	17,077	5.2	(5.1–5.3)
Overweight	113,720	74.5	(74.2–74.7)	28,552	18.7	(18.5–18.9)	10,438	6.8	(6.7-7.0)
Obesity	76,001	69.0	(68.7–69.2)	22,607	20.5	(20.3–20.8)	11,588	10.5	(10.3–10.7)
**Maternal age at hospital admission**									
< 20	6,212	65.5	(64.6–66.5)	1,712	18.1	(17.3–18.8)	1,556	16.4	(15.7–17.2)
20–24	41,105	72.0	(71.6–72.4)	10,008	17.5	(17.2–17.8)	5,977	10.5	(10.2–10.7)
25–29	121,532	75.2	(75.0-75.4)	28,551	17.7	(17.5–17.8)	11,583	7.2	(7.0-7.3)
30–34	196,236	76.6	(76.5–76.8)	45,120	17.6	(17.5–17.8)	14,687	5.7	(5.6–5.8)
35–39	106,936	75.7	(75.5–76.0)	26,246	18.6	(18.4–18.8)	7,996	5.7	(5.5–5.8)
≥ 40	22,981	73.1	(72.6–73.6)	6,601	21.0	(20.5–21.4)	1,868	5.9	(5.7–6.2)
**Parity**									
Multiparity	314,643	75.6	(75.5–75.8)	69,392	16.7	(16.6–16.8)	32,017	7.7	(7.6–7.8)
Primiparity	180,359	74.9	(74.7–75.1)	48,846	20.3	(20.1–20.4)	11,650	4.8	(4.8–4.9)
**Multiple pregnancy**									
Yes	9,324	47.3	(46.6–48.0)	8,746	44.3	(43.6–45.0)	1,654	8.4	(8.0-8.8)
No	485,678	76.2	(76.1–76.3)	109,492	17.2	(17.1–17.3)	42,013	6.6	(6.5–6.7)
**Maturity status**									
Term	474,402	77.0	(76.9–77.1)	101,637	16.5	(16.4–16.6)	39,753	6.5	(6.4–6.5)
Preterm	20,600	50.1	(49.6–50.6)	16,601	40.4	(39.9–40.9)	3,914	9.5	(9.2–9.8)
**Delivery mode**									
Cesarean section	140,376	67.1	(66.9–67.3)	49,799	23.8	(23.6–24.0)	19,133	9.1	(9.0-9.3)
No cesarean section	354,626	79.2	(79.1–79.3)	68,439	15.3	(15.2–15.4)	24,534	5.5	(5.4–5.5)
**Sex**									
Male	252,719	75.2	(75.0-75.3)	61,185	18.2	(18.1–18.3)	22,345	6.6	(6.6–6.7)
Female	242,176	75.6	(75.4–75.7)	57,020	17.8	(17.7–17.9)	21,314	6.7	(6.6–6.7)
Diverse	107	72.3	(65.1–79.5)	33	22.3	(15.6–29.0)	8	5.4	(1.8-9.0)
**Length of hospital stay**									
≤ 1 day	85,004	70.2	(69.9–70.4)	25,822	21.3	(21.1–21.5)	10,327	8.5	(8.4–8.7)
2–3 days	341,258	77.8	(77.7–78.0)	69,637	15.9	(15.8–16.0)	27,584	6.3	(6.2–6.4)
4–6 days	64,751	71.7	(71.4–72.0)	20,397	22.6	(22.3–22.9)	5,179	5.7	(5.6–5.9)
> 6 days	3,989	57.4	(56.2–58.6)	2,382	34.3	(33.2–35.4)	577	8.3	(7.7-9.0)
**Transfer to children’s hospital**									
Yes	31,050	51.4	(51.0-51.8)	23,290	38.5	(38.2–38.9)	6,082	10.1	(9.8–10.3)
No	463,952	77.8	(77.7–77.9)	94,948	15.9	(15.8–16.0)	37,585	6.3	(6.2–6.4)

95% CI, 95% confidence interval; BMI, Body mass index

Preterm newborns were less often exclusively fed with human milk, whereas the proportions of being partially fed with human milk or exclusively fed with formula were higher among preterm than term newborns. Newborns delivered vaginally were more frequently exclusively fed with human milk and less often partially fed with human milk or exclusively fed with formula than after caesarean section. Newborns with a hospital stay of one day or shorter or longer than four days were less often exclusively fed with human milk and more often partially fed with human milk or exclusively fed with formula than after a hospital stay of 2–3 days. Similarly, newborns transferred to a children’s hospital were less often exclusively fed with human milk and more often partially fed with human milk or exclusively with formula than children not transferred.

**Fig. 2:**
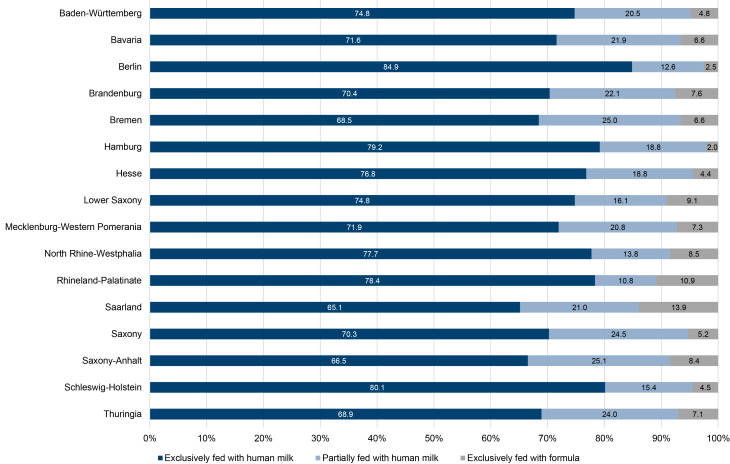
Prevalence rates of the newborn’s nutrition at hospital discharge or transfer according to federal state of the maternity hospital

There were regional differences across the 16 federal states of Germany with the highest proportion of children being exclusively fed with human milk in Berlin (84.9%, *n* = 31,846) and the lowest in Saarland (65.1%, *n* = 5,852), Saxony-Anhalt (66.5%, *n* = 8,979), and Bremen (68.5%, *n* = 5,192), whereas the proportions of children being exclusively fed with formula were lowest in Hamburg (2.0%, *n* = 478) and Berlin (2.5%, *n* = 939) and highest in Saarland (13.9%, *n* = 1,246) (Fig. 2). The proportions of children partially fed with human milk were highest in Saxony-Anhalt (25.1%, *n* = 3,384), Bremen (25.0%, *n* = 1,895), Saxony (24.5%, *n* = 6,348) and Thuringia (24.0%, *n* = 3,042), and lowest in Rhineland-Palatinate (10.9%, *n* = 3,161).

## Discussion

According to the nationwide German Perinatal Statistics, which since 2021 routinely collects information on newborn nutrition at discharge or transfer from the maternity hospital, nearly every fourth newborn in Germany was not exclusively fed with human milk at the time of leaving the maternity hospital. Of those, most were partially fed with human milk, and only a minority did not receive human milk at all. Newborns exposed to pre- and perinatal risk factors including maternal overweight or obesity, younger maternal age (< 25 years), multiple pregnancy, prematurity, or caesarian section had lower rates of being exclusively fed with human milk.

Despite the considerable number of newborns not being exclusively fed with human milk in the first days of life, the prevalence of newborns receiving human milk either exclusively or partially was high at 93%. In comparison to data from the cross-sectional nationwide studies KiGGS Wave 2 and “breast-feeding and infant nutrition in Germany” (SuSe II), the rate of ever breast-feeding (birth cohort 2013/2014) was 87% [13] and the rate of any breastfeeding (2017–2019) was 94% [28], respectively. The breastfeeding rates observed at discharge are also comparable to those in SuSe II: exclusive breastfeeding 74%, partial breastfeeding 20% (including predominant breastfeeding), 6% no breastfeeding [28]. However, comparability of both studies with our data is limited due to methodological differences, including the cohort-based design, potential participant selection bias and recall bias. Both studies also observed an increase in the prevalence of breastfeeding for Germany. In KiGGS, the proportion of children who were ever breastfed increased from 82.5% in Wave 1 (birth cohort 2007–2008) [29] to 87% in Wave 2 (birth cohort 2013–2014) [13]. Similarly, data from the SuSe studies showed an increase in any breastfeeding at hospital discharge from 86% in SuSe I (1997–1998) to 94% in SuSe II (2017–2019) [28]. An international comparison of rates of ever breastfeeding in high-income countries including data from 2001 to 2019 showed a median rate of 91% among countries, half of them reporting a prevalence rate of higher than 90% [30]. Thus, a positive trend in breastfeeding in Germany over the past decade might be assumed, in spite of limitations in comparability.

Nonetheless, the prevalence of exclusively feeding human milk at hospital discharge or transfer was only 75%. Among all newborns, 18% were fed with a combination of human milk and formula, despite evidence that introducing formula to a breastfeeding infant during the hospital stay can compromise the success of breastfeeding [31]. Women who fed their child a combination of human milk and formula in the hospital had an almost 3-times higher risk for breastfeeding cessation by nine weeks after delivery [31]. In our analysis, several groups appeared to be particularly vulnerable: the proportion of infants fed with a combination of human milk and formula was highest among multiple births as well as among preterm newborns, exceeding 40% in both groups. Furthermore, the highest rate of exclusive formula feeding and thus lower rates of exclusive breastfeeding were observed among younger mothers (< 25 years) and mothers with a BMI within the range of overweight or obesity compared to older mothers and mothers with normal weight. These findings are consistent with previous studies [29, 32, 33]. Reasons why women with overweight or obesity are less likely to exclusively breastfeed are presumed to be e.g. delayed lactogenesis and difficulties with effective milk drainage [34]. Therefore, promotion and support of exclusive breastfeeding by applying the “10 Steps to Successful Breastfeeding” in the first days after birth is important, since it is an effective measure to increase breastfeeding initiation, particularly in the identified vulnerable subgroups with high rates of partial breastfeeding and/or exclusively formula feeding (Supplementary Material 1) and will improve breastfeeding duration of any and exclusive breastfeeding [35]. Particularly, in the high proportion of newborns after cesarean section, a specific focus should be provided in guiding and supporting initiation of breastfeeding through e.g. skin-to-skin contact. In hospitals that apply the 10 Steps, promotion of breastfeeding has priority, and mother-child relationships are not disturbed e.g. by inappropriate routine postnatal practices [36]. However, after hospital discharge, an adequate follow-up care is essential, particularly in women who are discharged within 1–3 days postpartum, often before lactogenesis II is fully established, for exclusive breastfeeding to be achieved in the weeks after delivery.

A longer postnatal hospital stay or transfer of the newborn to the children’s hospital were associated with lower rates of exclusively receiving human milk, most likely explained by illnesses and separation of mother and child. Further results including lower rates of exclusively feeding human milk among women with primiparity or multiple pregnancy, in preterm newborns, or following cesarean section were consistent with results of previous studies [29, 37]. The percentage of preterm newborns included in the present analysis was lower than reported for all hospital births in Germany, as reported by the Perinatal Statistics (6.3% vs. 7.6%) [20]. As a reason, preterm newborns are often transferred to neonatal units immediately after birth and may therefore not be fully captured within the obstetric dataset; in these cases, information on newborn’s nutrition is documented within the Neonatology module of the Perinatal Statistics, which reflects their specific clinical care setting. These findings highlight the need for targeted interventions focusing on these groups to support and promote exclusive breastfeeding.

In an attempt to interpret regional differences, a first comparison of rates of newborns exclusively being fed with human milk and rates of deliveries in certified baby-friendly hospitals between the 16 different federal states was conducted. However, this comparison is subject to an important limitation, as it is based on different data sources; therefore, no direct relationships can be inferred from these findings, and it needs to be interpreted with caution, requiring future confirmation. Data on the number of deliveries in certified baby-friendly hospitals in 2021 were derived from the German WHO/United Nations Children’s Fund (UNICEF) Baby-Friendly Initiative on request. In most federal states of Germany, the proportions of newborns exclusively fed with human milk and of births occurring in baby-friendly hospitals tend to be either both above or both below the national average, suggesting concordant patterns; this is most evident in the federal state of Berlin (Supplementary Material 2). This trend was not observed in Baden-Württemberg, Hamburg, Hesse, Lower Saxony, Rhineland-Palatinate, and Saxony (in total 6 out of 16 federal states).

Currently, only 20% of all deliveries occur in certified baby-friendly hospitals in Germany [38], although it is possible that more hospitals apply the 10 steps in their practice but are not certified because the costs of certification must be covered by the hospitals. A nationwide implementation of the “10 Steps to Successful Breastfeeding” in all maternity and childbirth facilities, as one measure in the strategic field “Prevention and healthcare structures” of the German National Strategy for the Promotion of Breastfeeding [17, 18], may increase breastfeeding rates. However, evidence suggests that sustainable improvements require embedding these hospital-based measures within a broader support concept that begins during pregnancy and continues after discharge [39]. Federal states in Germany also differ in socioeconomic and demographic characteristics, including population composition, density, employment rates, and urban–rural distribution, which can influence breastfeeding practices. Recent evidence from North Rhine-Westphalia shows that hospitals in districts with higher average socioeconomic status are more likely to implement the 10 Steps, which in turn is associated with higher breastfeeding rates at discharge [40]. While the proportion of births in baby-friendly hospitals provides one possible explanation, other factors likely play a role, e.g. socioeconomic determinants or midwife coverage.

This study has strengths and limitations. A major strength of this study is the nationwide scope and large dataset, allowing the first comprehensive evaluation of a newly introduced indicator of newborn feeding practices in routine perinatal care in Germany. However, data on the newborn’s nutrition at hospital discharge or transfer were only available for 85% of children in the first year of implementation. The missing data are largely methodological in nature, possibly exacerbated by (1) pandemic-related structural changes in clinical workflows and widespread operational pressure on maternity services at that time [41] and (2) implementation problems – not primarily due to inadequate documentation. Consistent with this hypothesis, in subsequent years, fewer data were missing for this variable [42, 43]. According to the documentation rules, the variable was not mandatory for live-born newborns discharged or transferred within less than 4 h after birth. In practice, newborns who are transferred early because of illness and prematurity as well as infants with very rapid discharges after uncomplicated births may not be included in the dataset. While this may have introduced some bias, our data seem plausible and align with previous studies. There is no indication that the missing data were systematically biased, and we therefore do not expect the missing data to have affected the overall interpretation. Although it cannot be excluded that hospitals with a particular interest in breastfeeding support may have been more likely to document feeding practices at discharge, a substantial bias in this direction appears unlikely, given that feeding mode at discharge represents only one of the more than 100 data items routinely collected for the perinatal statistics database. However, analyses of the distribution of missing data e.g. across federal states may provide important insights for future secondary analyses. Furthermore, only data from hospitals and not data from birth centers could be considered. The instructions provided to hospital staff for completing the documentation form lacked precise definitions for predominant breastfeeding, potentially resulting in ambiguous classification. In addition, it was not specified whether the information reflect feeding practices throughout the entire hospital stay or, more likely, only to the day of discharge (i.e. the last 24 h). Therefore, the documentation instructions and definitions should be clarified and revised accordingly for future survey years.

## Conclusion

Nearly one in four newborns is not exclusively breastfed at discharge/transfer from the maternity hospital, underscoring the need to promote early breastfeeding initiation. Targeted support may be particularly beneficial for women with overweight/obesity, younger mothers (< 25 years), multiple pregnancies, prematurity, or after caesarean section. Wider implementation of the “10 Steps to Successful Breastfeeding” in maternity hospitals as one important measure of the *National Strategy for the Promotion of Breastfeeding* [18] could help increase breastfeeding rates, particularly among at-risk mother-child pairs given the need for resource allocation and prioritization of financial goals.

Beyond its descriptive findings, the present analysis has implications for the future use of newborn feeding indicators within the German Perinatal Statistics. It supports continued collection of data on newborn feeding at hospital discharge as part of routine quality assessment. Future evaluations could be expanded to include indicators proposed by the WHO [26], e.g. early initiation of breastfeeding within the first hour of life, ideally with more precise specification regarding newborn feeding during the final 24 h prior to discharge or transfer. Indicators such as exclusive or any breastfeeding beyond discharge need to be assessed through additional data collections.

The data provide a valuable foundation for breastfeeding monitoring and health reporting, as well as for future research examining associations between specific variables and newborn feeding outcomes. The extent to which specific maternal, neonatal, and contextual factors influence neonatal feeding outcomes should be addressed in additional projects, aiming at the identification of potential targets for interventions and research within a PDCA (Plan-Do-Check-Act) framework. Ongoing systematic collection and analysis of these data could inform public health strategies and enable evidence-based breastfeeding support policies.

## Electronic Supplementary Material

Below is the link to the electronic supplementary material.

Supplementary Material 1: Subgroups with rates of “partially fed with human milk“ of > 40% and rates of „exclusively fed with formula“ of > 9%

Supplementary Material 2: Prevalence rates of children exclusively fed with human milk and deliveries in certified baby-friendly hospitals across the federal states of Germany in 2021

## Data Availability

Data from quality assurance procedures pursuant to section  136 of the German Social Code, Book Five (Sozialgesetzbuch, SGB V) of the Federal Joint Committee (Gemeinsamer Bundesausschuss, G-BA) were used. The dataset analyzed during the current study are available on request from the IQTIG, [https://iqtig.org/qs-verfahren-uebersicht/sekundaere-datennutzung/].

## Abbreviations

BBF: Becoming Breastfeeding Friendly
BMI: Body mass index
BZfE: Federal Center of Nutrition
CI: Confidence interval
G-BA: Federal Joint Committee
IQTIG: Institute for Quality Assurance and Transparency in Healthcare
KiGGS Wave 2: German Health Interview and Examination Survey for Children and Adolescents Wave 2
MRI: Max Rubner-Institut
UNICEF: United Nations Children’s Fund
WHO: World Health Organization
