# Supplementary material for: Breastfeeding at discharge or transfer from the maternity hospital: results from the German Perinatal Statistics 2021
Source: Int Breastfeed J. 2026 Jun 23;21:59. doi: 10.1186/s13006-026-00862-5 (PMC13295186; doi:10.1186/s13006-026-00862-5)
Supplement: Supplementary file 1 — Supplementary Material 1: Subgroups with rates of “partially fed with human milk“ of > 40% and rates of „exclusively fed with formula“ of > 9% [file 13006_2026_862_MOESM1_ESM.docx]

**Supplementary Material 1:** Subgroups with rates of “partially fed with human milk“ of > 40% and rates of „exclusively fed with formula“ of > 9%
**bold** = “partially fed with human milk“ > 40% or rates of „exclusively fed with formula“ > 9%
