# Supplementary material for: Breastfeeding at discharge or transfer from the maternity hospital: results from the German Perinatal Statistics 2021
Source: Int Breastfeed J. 2026 Jun 23;21:59. doi: 10.1186/s13006-026-00862-5 (PMC13295186; doi:10.1186/s13006-026-00862-5)
Supplement: Supplementary file 2 — Supplementary Material 2: Prevalence rates of children exclusively fed with human milk and deliveries in certified baby-friendly hospitals across the federal states of Germany in 2021 [file 13006_2026_862_MOESM2_ESM.docx]

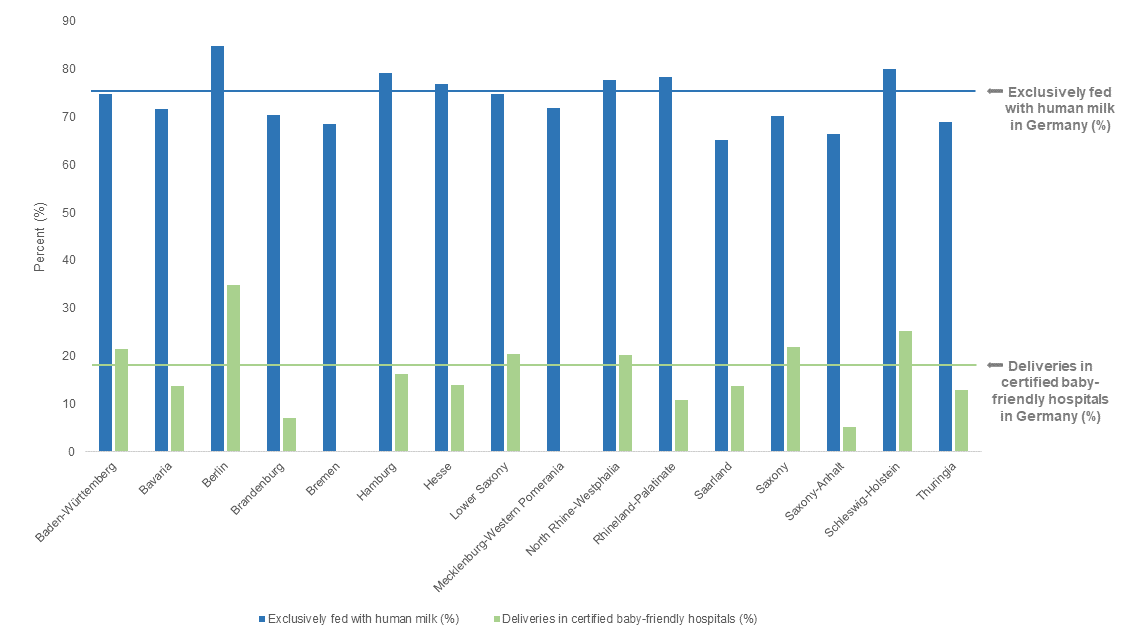
**Supplementary Material 2:** Prevalence rates of children exclusively fed with human milk and deliveries in certified baby-friendly hospitals across the federal states of Germany in 2021
